# Making Cognitive Niches Explicit: On the Importance of External Cognitive Representations in Accounting for Cumulative Culture

**DOI:** 10.3389/fnint.2021.734930

**Published:** 2021-10-27

**Authors:** Mateusz Hohol, Kinga Wołoszyn, Bartosz Brożek

**Affiliations:** ^1^Copernicus Center for Interdisciplinary Studies, Jagiellonian University, Kraków, Poland; ^2^Institute of Psychology, Jagiellonian University, Kraków, Poland; ^3^Faculty of Law and Administration, Jagiellonian University, Kraków, Poland

**Keywords:** cognitive artifacts, cognitive history, cumulative culture, representation, wide cognition

## Abstract

Cumulative transmission and innovation are the hallmark properties of the cultural achievements of human beings. Cognitive scientists have traditionally explained these properties in terms of social learning and creativity. The non-social cognitive dimension of cumulative culture, the so-called technical reasoning, has also been accounted for recently. These explanatory perspectives are methodologically individualistic since they frame cumulative and innovative culture in terms of the processing of inner cognitive representations. Here we show that going beyond methodological individualism could facilitate an understanding of why some inventions are disseminated in a stable form and constitute the basis for further modifications. Drawing on three cases of cognitive history of prominent achievements of Antiquity, i.e., Homerian epics, Euclidean geometry, and Roman law, we investigate which properties of cognitive artifacts shaped cognitive niches for modifying original cognitive tasks or developing new ones. These niches both constrained and enabled the cognitive skills of humans to promote cumulative culture and further innovations. At the same time, we claim that “wide cognition,” incorporating both intracranial resources and external cognitive representations, constitutes a platform for building explanations of cognitive phenomena developing over a historical time scale.

## Introduction

The most prominent cultural enterprises of humans, such as literature, mathematics, and civil law, are too complex to be the products of single persons. Instead, they are deeply social, resulting from collective efforts involving many individuals. In the broadest perspective, these enterprises are thought to be driven by two engines, namely, transmission and innovation (Legare and Nielsen, 2015). The former involves the gradual accumulation of practices and elements of material culture, while the latter introduces something new or helps to refine existing resources.

Cultural transmission is primarily investigated in terms of social learning, especially active teaching and imitation, working in concert with motivational mechanisms and collaborative attitudes (Iacoboni, 2009; Tomasello, 2009; Jenson and Iacoboni, 2011; Heyes, 2018). Introducing innovation is genuinely accounted for in terms of trial-and-error learning, supported by metacognition (Sawyer, 2011; Abraham, 2013). This kind of dualism in the foundations of culture has been recently challenged by Osiurak and Reynaud (2020). They proposed that the mechanisms of social learning only “catalyze” technical reasoning skills that enable individuals to both innovate and disseminate material culture in a cumulative way.

Despite their differences, both of these approaches to culture, i.e., social learning accounts and technical-reasoning hypothesis, are methodologically individualistic. They focus on the capacities of individuals, being elucidated in terms of their skull-bounded cognitive systems. This is clear in the case of the technical-reasoning hypothesis (Osiurak and Reynaud, 2020), but also the most recent social learning accounts, such as Heyes’s (2018) cultural evolutionary psychology, are still committed to methodological individualism (Baggs et al., 2019). Here we propose that going beyond methodological individualism and focusing more on properties of external cognitive representations (called “cognitive artifacts”) might facilitate answering the question of why some inventions are disseminated in a stable form and constitute the basis for further modifications. From a more general perspective, an anti-individualist approach to cognition could build bridges between research in mechanistic cognitive science and historically oriented humanities.

The paper proceeds as follows. First, we outline the transition of cognitive science from methodologically individualistic research to studying “wide” cognitive systems involving multiple individuals and cognitive artifacts. We then turn to a discussion of the notion of cognitive artifact to justify that objects external to the human body could enhance cognition. Next, we introduce three case studies from cognitive history, namely Homerian epics, Euclidean geometry, and Roman law, to investigate which properties of cognitive artifacts promoted cumulative culture. While these enterprises were based on different types of artifacts, our focus is on language-based artifacts. Finally, we propose future research directions.

## From Individual Minds to Cognitive Niches

Although pioneers of sociocultural psychology emphasized the prominent role played by society and culture in shaping human cognition (e.g., Vygotsky, 1934/1986), their contribution was mostly ignored in behaviorism and early cognitive science (see Norman, 1991). The latter enterprise traditionally focused on the cognition of individuals, sandwiched between the brain systems responsible for perception and action, and accounted for in terms of computing internal representational states (Fodor, 1975; Newell and Simon, 1976). This kind of methodological individualism led to humans’ natural and artificial surroundings being systematically neglected or at least underestimated in studies on cognition (Norman, 1991).

In the 1980s, the situation began to change, and cognitive science focused on the brain and the environment (Bechtel et al., 1998). At that time, embodied cognition, a research program emphasizing that the mind is deeply anchored in the sensorimotor systems, came to the fore (Lakoff and Johnson, 1980). In the next decade, culturally oriented psychology (Cole, 1996) was reborn and emphasized the deeply social nature of humans (see Tomasello, 2009; Brożek, 2013). The focus of cognitive science on embodiment and enculturation provided the impetus for further studies within the framework of such positions as embedded cognition, extended mind, enactivism (called “4E cognition”; Newman et al., 2018) and distributed cognition (Hutchins, 1995). Miłkowski et al. (2018) recently put all of them under the umbrella term of “wide cognition,” and showed that when *taken together* they could be included in mechanistic explanations of cognitive phenomena.

The “wide” account is anti-individualistic since it assumes that interactions and artifacts do not only trigger cognitive processes running in the individual’s head (or body) but also serve as constituents of cognition. According to this position, in many cases, we cannot identify the center of the cognitive system within an individual human being (Hutchins, 2014). Probably the most discussed example is a marine navigation system before the GPS era. Hutchins (1995) claims there is no single officer on board the ship who stores and manipulates all information relevant to navigation. According to the researcher, the cognitive system revealing the capacity to navigate consists of many individuals who interact with instruments via their sensorimotor systems, wherein a map is the center of the whole system. Here, the focus only on internal processes (occurring in the brains of individuals) is not sufficient to comprehensively explain the phenomenon of interest. Importantly, Hutchins (1995, 2014) identifies cognition with the processing (sequential or simultaneous) of representational states anchored in heterogeneous components. These states could include spoken or written linguistic expressions, gestures, information embedded in a chart or a map, etc. Although one could argue that all of them are human-made (in one way or another) and thus grounded in internal processes, the difference between methodological individualism and the “wide” approach is not a difference of type but a difference of the level of the analysis.

Thus, in a nutshell, the “wide” account frames cognition as non-trivially grounded in sensorimotor activity and at the same time being dynamically reshaped and extended in interactions with other individuals and components of the surrounding. Reshaping and extending cognition takes place through the processes of constructing cognitive niches. Here, the term “constructing” refers to a particular theoretical approach to cognitive niches. For Pinker (2010), “cognitive niche” is a uniquely human and general “mode of survival characterized by manipulating the environment through causal reasoning and social cooperation” (p. 8993) that was achieved by *Homo sapiens* at some period of evolutionary history (see Bertolotti and Magnani, 2017). Here, however, we endorse Clark’s (2006) constructionist approach, assuming that a cognitive niche is “an animal-built physical structure that transforms one or more problem spaces in ways that (when successful) aid thinking and reasoning about some target domain or domains. These physical structures combine with appropriate culturally transmitted practices to enhance problem-solving and (in the most dramatic cases) to make possible whole new forms of thought and and reason” (p. 370). According to this account, and contrary to Pinker (2010), there is no single cognitive niche of the human being (for comparison of both approaches, see Bertolotti and Magnani, 2017). Instead, collaborating individuals construct their own cognitive niches locally through employing external objects, called cognitive artifacts.

## Can Artifacts Be Cognitive Representations?

Clark’s approach to cognitive niches (and “wide cognition” in general) assumes that artifacts in cognitive science, indeed, play a role similar to that traditionally attributed to internal mental representations. Before discussing properties of particular constituents of cognitive niches that allowed the development of Homerian epics, Euclidean geometry, and Roman law, we will investigate the notion of “cognitive artifact.”

According to Norman (1991), cognitive artifacts are “artificial devices designed to maintain, display, or operate upon information in order to serve a representational function and that affect human cognitive performance” (p. 17; see Hutchins, 1999; Brey, 2005). In other words, they are human-made entities contributing functionally to improving cognition thanks to representing something. This broad class includes timetables, calendars, or checklists, when aiding our memory (Clark, 2003), and maps and compasses, when supporting our spatial navigation (Hutchins, 1995). Words, phrases, diagrams, and other symbols embedded in paper and other media also belong to the category of cognitive artifacts when they facilitate the making of inferences.

Given this diversity, taxonomies of cognitive artifacts have been proposed. For instance, Brey (2005) distinguished classes of artifacts relying on the domains of cognition that they enhance (e.g., memory, interpretation, search). In turn, Heersmink (2013) distinguished two main classes: *representational cognitive artifacts*, that contain “information about the world” (p. 476), and so-called *ecological cognitive artifacts*, which serve “as the world” (ibid.). The former includes maps, compasses, or abacuses. An example of the latter, namely, ecological one, is the spatial arrangement of objects to prioritize tasks to do (see also Kirsh, 1995).

Amongst others, Heersmink (2013) questioned Norman’s (1991) assumption that performing a representational function by an artifact is necessary to classify it as cognitive one. Many other theorists, particularly those sympathizing with dynamical accounts of cognition, support non-representational approaches to artifacts (Rączaszek-Leonardi et al., 2019). On the other hand, Heersmink’s (2013) proposal of the existence of ecological cognitive artifacts has been challenged (Fasoli, 2018). Prioritizing tasks through arranging space could be easily reinterpreted, e.g., the spatial order of printed papers might “stand behind” the information flow relevant to scientific enquiry. Thus, “all cognitive artifacts are, in different ways, representational” (Fasoli, 2018, p. 6).

Although maps are frequently considered prototypical cognitive artifacts, it is not obvious what *contributes* functionally to navigation: the material object held in hands, its high-level cognitive representation, or the low-level brain activity triggered by the map. This example shows that cognitive artifacts inherit some of the more general problems of functionalism (Vaccari, 2017). To solve those problems, Fasoli (2018) has recently proposed an alternative, interaction-centered approach to cognitive artifacts, while simultaneously introducing a novel taxonomy. For him, cognitive artifacts are “those physical objects that have been created or modified to contribute to the completion of a cognitive task, providing us with representations that we employ for substituting, constituting or complementing our cognitive processes, thus modifying the original cognitive task or creating a new one” (ibid., p. 11). In this approach, a map is an example of *complementary cognitive artifact*, since it complements a cognitive process, i.e., spatial cognition, which can occur without it. In turn, another device supporting our place-finding, GPS, belongs to a category of *substitutive cognitive artifacts* since it replaces navigating with “inner spatial sense” by relying on cues delivered by the device. The final category involves *constitutive cognitive artifacts*. They are necessary to complement cognitive tasks that could not exist without them. Text is a primary example of this category. No matter what media a text is saved or stored in, reading is impossible without it.

One can make an analogous distinction to the one between a map and GPS with an abacus (it complements numerical cognition processes) and a calculator (it substitutes these processes). However, the abacus is a relatively new artifact, most probably invented by the Greeks in the seventh century BC (Sugden, 1981), while linguistic expressions inscribed on clay tablets and written on papyrus are much earlier. Although the ancient Egyptian symbolic systems were not convenient for performing calculations, the pre-linguistic capacity of a human being to cope with numerical magnitudes would be most likely too limited to establish more advanced forms of numerical cognition without them (Spelke, 2013). In other words, language is a constitutive artifact for mathematics, yet this does not exclude the possibility that language could be further complemented/substituted with other artifacts. This example highlights that, in order to understand the contribution of external cognitive representations to our cultural achievements, taxonomical work should be accompanied by the investigation of the cognitive history of artifacts.

## Three Case Studies in Cognitive History

Cognitive history is a field that combines historical research and cognitive science in order to elucidate the origins of particular forms of human knowledge, thinking, and communication (e.g., Netz, 1999; Sutton and Keene, 2016; Dunér and Ahlberger, 2019). While focusing on artifacts, cognitive history “approaches knowledge not through its specific propositional contents but through its forms and practices” (Netz, 1999, p. 7). In this section, we will discuss three cases of cognitive niches that enabled prominent achievements of Antiquity, namely, Homerian epics, Euclidean geometry, and Roman law. In particular, we will indicate the language-based cognitive artifacts that, respectively, aided extraordinary mnemonics, facilitated the development of deductive proofs, and enabled normative thinking.

### Homeric Epics: Toward Extraordinary Mnemonics

Most likely, Homeric epics were composed and initially transmitted by illiterate improvisers. Considering the limited memory capacity of humans, the question arises as to how the relatively faithful transmission of groups of long phrases was cognitively possible in the absence of written form. The Parry–Lord theory delivers a promising answer (Parry, 1930; Lord, 1960). It states that illiterate improvisers relied on oral *formulae*, namely, fixed strings of words “regularly employed under the same metrical conditions to express a given essential idea” (Parry, 1930, p. 80). The formula could be classified here as a special case of the language-based cognitive artifact—it is a human-made linguistic pattern manifested in the acoustic space that enhances cognition by complementing memory. In this scenario, memory capacities’ extension proceeded by memorizing a limited set of ready-made formulae and acquiring procedural knowledge about metrical conditions. Having these “tools” to hand, improvisers could place fixed strings of words into the prosodic slots of the verse to both introduce innovation and disseminate it faithfully.

Constraining the language of poetry was most likely a gradual process made without any power relationships. The cognitive niche for Homeric epics was created thanks to interactions between improvisers and their audience “glued” by a particular type of language-based cognitive artifact. Summing up, the cognitive niche for Homeric poetry transformed a hard problem (how to remember large portions of language expressions exactly) into a simpler one (how to remember small portions of formulae and combine them under metrical conditions). The success of Homeric improvisers, despite their lack of literacy, ceases to be puzzling since formulae are easy to share thanks to their relative simplicity and grounding in cognitive capacities common to human beings: linguistic skills and a sense of rhythm.

### Greek Geometry: The Birth of Proof

Shaping a new cognitive artifact by constraining linguistic practices could also be found outside of Homerian epics. The development of ancient mathematics could be considered a transition from recipes for solving practical problems in concrete cases to appreciating “the power of proof,” connected with recognizing the necessity and generality of reasonings performed with abstract concepts. The former state was characteristic for Egyptian and Babylonian mathematics, while the latter—for Greek geometry with Euclid’s “Elements” to the fore (Russo, 2004). Although “the power of proof” is typically considered in the context of artificial symbols of modern mathematics, the representational layer of Greek geometry completely lacks this kind of resource. The question which arises is how the Euclidean proof was cognitively possible at all.

In his detailed investigation on the cognitive history of geometry, Netz (1999) argues that two details of Greeks’ cognitive artifacts, basically absent in Egypt and Babylonia, allowed the success of deductive mathematics. The first was the fact that the language of mathematics was constrained in terms of its small manageable lexicon and limited combinations of elements. The second was marking the points of a diagram with letters to connect it with the textual part of the discourse rigidly. Here we focus only on the language (for diagrams see Hohol and Miłkowski, 2019; Hohol, 2020; Netz, 2020).

Discussing the linguistic constraints of geometry, Netz (1999) applies the term of *formula*, wherein, unlike Homerian epics, fixed patterns were written and independent from meter. An availability for transformation is their crucial feature. For Netz, formulae have a generative structure “in the sense that new expressions may be combined by fitting new formulae in the allowed slots” (p. 156). Accordingly, “…there is a system of rules for the production of recognizably formulaic structures” (ibid.). This kind of encapsulated computational system allows manipulations to no worse a degree than in systems built upon modern artificial symbols. What is more, formulae stabilized geometric practice by removing the ambiguities of daily communication. Within geometric language that constrains reasonings, the same manipulations on formulae always lead to the same results. Furthermore, a limited lexicon and relatively small set of formulae facilitate the acquisition of geometry, orientation in the logical structure of a problem, and ultimately the transfer of elements of the reasoning to further geometric problems. The latter feature clearly favors mathematical innovations. Regarding the social component of the cognitive niche for geometry, there was no power relationship or official curricula in ancient Greece that could dictate how to represent geometric problems. The anecdotes about mathematicians working in solitude are also far from historical evidence. Instead, Netz (1999) claims that progress in mathematics was made thanks to the joint efforts of multiple amateurish autodidacts who formed spontaneous social networks. This collaborative work gradually evolved from everyday language communication to encapsulating geometric discourse into formulae.

### Roman Law: From Descriptions to Norms

Early Roman law was highly casuistic, with legal decisions not based on general rules but better described as a particular reaction to concrete cases. However, even at this relatively early stage of development, a hearing before a private judge nominated by the *magistratus* began with “an outline of the essence of the case” called *causa coniectio* (Kupiszewski, 2013, p. 200). These succinct descriptions constituted cognitive resources that helped the judge to understand the adjudicated case.

It is argued that, with time, *causae coniectio* became *regulae iuris*, i.e., according to the definition given by Paulus, “something which briefly describes how a thing is” (Atria, 2001, p. 151). Paulus underscores that “the law may not derive from a rule, but a rule must arise from the law as it is” (ibid.). In other words, *regulae iuris* served as tools for organizing legal knowledge and facilitating the process of learning law and applying it. Importantly, *regulae* used quite general and abstract language.

In the beginning, *regulae* had no normative force. The problem is that it is difficult to distinguish a normative statement from a simple synthetic description and doing this requires extraordinary methodological discipline. What might have been natural and easy for a lawyer of the late Republic or early Empire would not be that obvious in later periods, especially in the Middle Ages, when *ius commune* began to develop, partly under the influence of canon law (Cairns and du Plessis, 2010). Over time, the *regulae* acquired normative power. This shift has opened the way for the emergence of the idea of a codified legal system consisting of abstract rules.

This historical trajectory of *regulae iuris*—from *causae coniectio*, through a methodologically conceived tool for understanding and teaching law, to normative statements—is quite illuminating. It points to a natural drift toward abstraction as well as the need to break away from casuistic court decisions and to search for patterns in the complex labyrinth of Roman law. And this tendency is hardly surprising: our minds find it easier to solve problems on the basis of a relatively small set of comparatively abstract rules than to apply a multitude of highly concrete normative precepts. An abstract system of legal rules is a cognitive artifact that is easier to transfer through social learning. Moreover, such a system provides us with a more coherent view of the “legal world,” enabling us to see similarities between distinct classes of cases and thus driving legal innovation (see Brożek, 2020).

## Conclusion and Future Research Perspectives

In this paper, we claimed that focusing on cognitive niches emerging from interactions between individual and cognitive artifacts could help build comprehensive explanations of the most intriguing properties of human culture, specifically, innovation and transmission. The potential advantage of this approach over traditional methodological individualism in cognitive science is at least twofold. First, it is recognized that focusing only on internal mental representations did not lead to building thoroughly integrative “the mind’s new science” (see Núñez et al., 2019). A symptom of this state has been the long-term marginalization of anthropology, which was assumed to be one of the pillars of cognitive science. The “wide” approach opens cognitive science to an anthropological perspective, which is essential for understanding the culture. Second, focusing on cognitive niches offers an opportunity to investigate the plasticity of human cognition that could be transformed by systematically creating and using new environment elements (see Hayles, 2012).

In closing, we would like to highlight some directions for future research. A few years ago, Hutchins noted that “the interesting questions concern the elements of the cognitive systems, the relation among the elements, and how cognitive processes arise from interactions among these elements” (Hutchins, 2014, p. 36). This could be read as a call to build more detailed mechanistic explanations of complex cognitive systems involving artifacts. For now, we have at our disposal only very rough sketches of such “wide” mechanisms (Miłkowski et al., 2018). Moreover, although more emphasis on cognitive history might be an impulse to integrate the research efforts of adepts of cognitive science and the humanities, a mechanistic reconstruction of past cognitive practices is a very risky task. Building a framework of methodological constraints for further attempts would be more than welcome. One of the potential risks relates to focusing on high-level interactions with others and artifacts while at the same time underestimating neuroscientific research. Theorists of wide cognition and cognitive artifacts should go deeper into the brain, and include also lower-level components in building their explanations. Methods of cognitive archeology could be useful for this task. For instance, similarly to cognitive archeologists who investigate the psychological and neural foundations of learning to prepare stone tools (see Malafouris, 2010 for review), researchers of language-based cognitive artifacts could study how written formulae, compared to more arbitrary linguistic expressions, affect the learning of mathematics.

## Data Availability Statement

The original contributions presented in the study are included in the article/supplementary material, further inquiries can be directed to the corresponding author/s.

## Author Contributions

MH, KW, and BB reviewed the literature, developed the theoretical stance, line of argument, wrote the manuscript, reviewed, revised, and accepted its final version. All authors contributed to the article and approved the submitted version.

## Conflict of Interest

The authors declare that the research was conducted in the absence of any commercial or financial relationships that could be construed as a potential conflict of interest.

## Publisher’s Note

All claims expressed in this article are solely those of the authors and do not necessarily represent those of their affiliated organizations, or those of the publisher, the editors and the reviewers. Any product that may be evaluated in this article, or claim that may be made by its manufacturer, is not guaranteed or endorsed by the publisher.
